# The Efficacy of Sodium-Glucose Cotransporter-2 Inhibitors in Improving Morbidity and Mortality of Heart Failure: A Systematic Review

**DOI:** 10.7759/cureus.34942

**Published:** 2023-02-13

**Authors:** Suvedha Thiagaraj, Twisha S Shukla, Sai Dheeraj Gutlapalli, Hadi Farhat, Huma Irfan, Kanmani Muthiah, Namratha Pallipamu, Sogand Taheri, Safeera Khan

**Affiliations:** 1 Internal Medicine, California Institute of Behavioral Neurosciences & Psychology, Fairfield, USA; 2 Pediatrics, California Institute of Behavioral Neurosciences & Psychology, Fairfield, USA; 3 Research, California Institute of Behavioral Neurosciences & Psychology, Fairfield, USA; 4 Internal Medicine, University of Balamand, Beirut, LBN; 5 Neurology, California Institute of Behavioral Neurosciences & Psychology, Fairfield, USA; 6 Internal Medicine, Franciscan Health, Lafayette, USA; 7 Medical Science, California Institute of Behavioral Neurosciences & Psychology, Fairfield, USA

**Keywords:** myocardial infarction, cardiovascular disease, sglt2 inhibitor, coronary artery disease, empagliflozin, dapagliflozin, canagliflozin, diastolic heart failure, systolic heart failure

## Abstract

Cardiovascular disease (CVD) is the leading cause of mortality in patients with type 2 diabetes mellitus (DM) worldwide. Sodium-glucose cotransporter-2 inhibitors (SGLT2i) were initially developed for treating patients with type 2 DM. The four major drugs developed are canagliflozin, dapagliflozin, empagliflozin, and ertugliflozin. Apart from treating DM, these drugs have shown to have a beneficial effect on lowering cardiovascular death and lowering hospital admission, and have beneficial renal outcomes. Recently, several large-scale randomized controlled trials (RCTs) were done to assess the benefit of these drugs, mainly in patients with CVD, irrespective of their diabetic status.

This systematic review examined seven large-scale randomized controlled trials that focused mainly on CVD in patients with type 2 DM and if it showed any improvement. We properly screened the RCTs if they demonstrated cardiovascular outcomes after taking the SGLT2i or a placebo drug. The seven studies combined had a total sample population of 55,433, and the mean follow-up time was about four years. The participants included in this study had various basal metabolic indices, ages, glomerular filtration rates, and diabetic status characteristics. Although these patients were quite different, after the administration of SGLT2i, the studies showed a beneficial effect in reducing CVD mortality and morbidity in patients with type 2 DM.

## Introduction and background

Diabetes mellitus (DM) is one of the most prevalent diseases globally; in the next few decades, the cases of DM worldwide will substantially increase to around 642 million by the year 2020, affecting the age group of 18-99 years [1]. Type 2 DM is on the list as the seventh cause of death worldwide, as it is a global pandemic, affecting millions of people in the world [2]. Every year, the fatalities linked to DM are estimated to be approximately around 1.6 million [3]. DM is slowly rising to be the leading global health problem as a chronic noncommunicable disease (CNCD) affecting people of different geographic, racial, and ethnic origins. Its prevalence is increasing slowly [4,5].

A disruption in carbohydrate, protein, and fat metabolism occurs due to a lack of insulin in the body, which leads to the development of DM [3]. DM results from a defect in insulin secretion or defect of insulin action, or both [4]. Patients with DM have a higher risk of developing heart failure (HF) compared to patients without DM [6]. The pathogenesis of DM is due to many crucial environmental factors such as diet, smoking, obesity, genetics, air pollution, physical inactivity, and stress [7]. DM presents various symptoms and comorbidities such as hyperglycemia, polyuria, polyphagia, polydipsia, vision changes including blurred vision, Kussmaul breathing, nausea, vomiting, altered consciousness, and abdominal pain. Presenting comorbidities include hypertension, heart failure, chronic kidney disease, and cardiovascular disease (CVD) [8].

There have been various drugs available for the treatment of DM, such as metformin, sulfonylureas, and insulin, for the past several decades. However, in recent times, there has been an emergence of newer drugs, such as glucagon-like peptide (GLP)-1 receptor agonists, dipeptidyl peptidase (DPP)-4 inhibitors, and sodium-glucose cotransporter-2 inhibitors (SGLT2i) [9]. Cardiovascular diseases (CVD) such as heart failure, myocardial infarction, and stroke are the most common cause of morbidity in patients with type 2 DM worldwide [10]. CVD has also become an emergent and leading health problem worldwide [10].

SGLT2i are a new class of drugs that help lower blood glucose levels. Several randomized controlled trials (RCTs) have investigated the effect of SGLT2i receptor agonists in reducing cardiovascular mortality and morbidity [11]. These drugs have recently improved cardiovascular outcomes and prevented the exacerbation of heart failure. SGLT2i decrease renal glucose reabsorption, thereby increasing the glucose excretion in the body, which considerably reduces the serum glucose level. Four SGLT2i have been approved by the United States Food and Drug Administration. These drugs are empagliflozin (Jardiance), canagliflozin (Invokana), dapagliflozin (Farxiga), and ertugliflozin (Steglatro) [12]. We mainly focused in this systematic review on discussing the cardioprotective benefits of SGLT2i in reducing morbidity and mortality in patients with heart failure irrespective of the diabetic status. The use of SGLT2i can become part of a guideline in the future to benefit patients. In this systematic review, we aim to focus on the effects of SGLT2i, mainly to see if they can improve the outcome of heart failure and other CVD based on the previously conducted major randomized controlled trial. 

## Review

Methods

This systematic review design and its results were conducted and reported in accordance with the Preferred Reporting Items for Systematic Reviews and Meta-Analyses (PRISMA) 2020 guidelines [13]. We strictly adhere to this method and its principles. The research question was formulated to identify if SGLT2i affected mortality and morbidity in heart failure patients.

Search Sources and Search Strategy

We used three major research literature electronic databases to search and extract our relevant articles thoroughly. To collect our data, we used PubMed, PubMed Central (PMC), and Cochrane Library from January 2 to January 9, 2022.

We generated suitable keywords using the Medical Subject Headings (MeSH) strategy and Boolean operators to identify the required articles. These keywords allowed us to accurately extract the necessary and relevant articles that demonstrate the evidence of the efficacy of SGLT2i in heart failure. These keywords were used separately and combined to find our articles in PubMed Central and Cochrane Library. Our keywords include “Sodium-Glucose Cotransporter-2 Inhibitors, Canagliflozin, Dapagliflozin, Empagliflozin, and Ertugliflozin, Systolic and Diastolic Heart Failure, Coronary Artery Disease, and Myocardial Infarction.”

The MeSH strategy that we generated and used in PubMed was “Sodium-Glucose Transporter 2 Inhibitors/Therapeutic Use” (Majr) AND (“Heart Failure/Drug Therapy” (MeSH) OR “Heart Failure/Mortality” (MeSH) OR “Heart Failure/Therapy” (MeSH)) through which a total of 260 articles were generated in PubMed.

Study Selection and Eligibility Criteria

Each article was then rigorously screened to remove duplicates, and the selected articles were thoroughly checked to illuminate irrelevant ones by going through the abstract, title, and subject headings. Then, each of the chosen articles was assessed for quality using the PRISMA checklist 2020 [13], and the full text was thoroughly read to satisfy the selection criteria. Then, the inclusion and exclusion criteria were applied to narrow down the selection of the article before we began the analysis. The entire systematic review was done under ethical boundaries.

Inclusion Criteria

We included human study articles published in the last five years in English literature. Then, we identified papers on the adult population related to systolic and diastolic heart failure and cardiovascular death and added papers related to SGLT2i. The patient, population, problem, intervention/exposure, comparison, and outcome (PICO) study criteria were the fulcrum behind the eligibility criteria.

Exclusion Criteria

**Figure 1:**
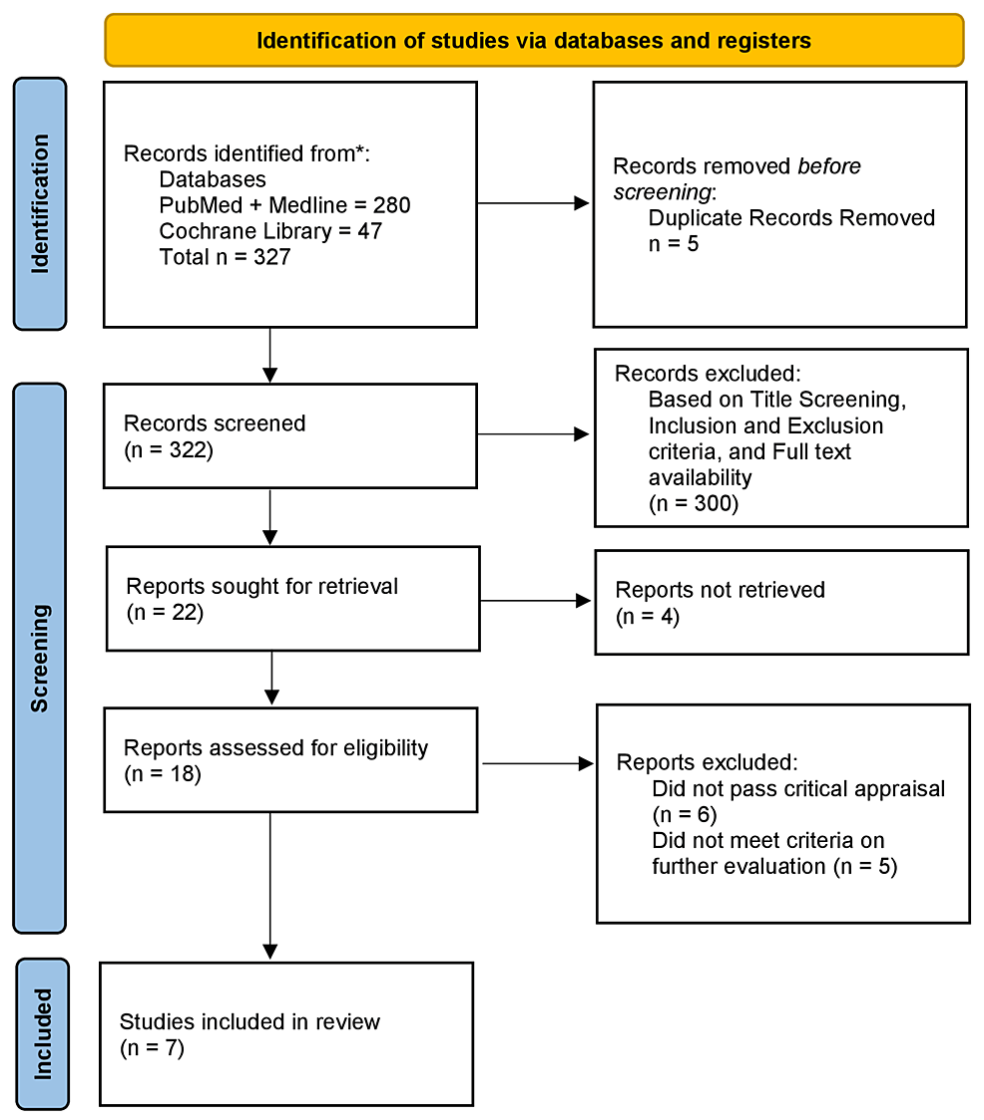
PRISMA diagram detailing the study identification and selection process PRISMA, Preferred Reporting Items for Systematic Reviews and Meta-Analyses

We decided to exclude the following from our paper: grey literature, letters to the editor, animal studies, unpublished literature, and papers discussing the pediatric population (Figure 1).

Risk of Bias and Quality Assessment 

All articles are independently assessed to decrease the risk of bias; we assessed every article’s quality using the Cochrane bias assessment tool for randomized controlled trials. We evaluated each potential cause of bias by rigorously checking each article for the seven domains random sequence generation, allocation concealment, blinding of participants, blinding of outcome assessment, incomplete outcome data, selective reporting, and other bias, based on low risk of bias, high risk of bias, and unclear risk of bias (Table 1).

**Table 1:** Cochrane appraisal +, low risk of bias; -, high risk of bias; ?, unclear risk of bias; N/A, not assessed

Cochrane appraisal	Random sequence generation	Allocation concealment	Blinding of participant	Blinding of outcome assessment	Incomplete outcome data	Selective reporting	Other bias
Packer et al., 2020 (EMPEROR-REDUCED) [14]	+	+	+	+	+	+	N/A
Cannon et al., 2020 (VERTIS CV) [15]	+	+	+	-	+	+	N/A
Petrie et al., 2020 (DAPA-HF) [16]	+	+	+	+	+	+	N/A
Perkovic et al., 2019 (CREDENCE) [17]	+	+	+	+	-	+	-
Wiviott et al., 2018 (DECLARE TIMI-58) [18]	+	+	+	+	+	+	+
Neal et al., 2017 (CANVAS) [19]	+	+	-	+	+	+	+
Wanner et al., 2016 (EMPA-REG) [20]	+	-	+	+	+	+	+

Results

After searching through many different online databases and libraries, we selected 327 articles to be reviewed for our study. We then screened the articles for duplicates and applied the inclusion and exclusion criteria that we formulated (papers related to SGLT2i and papers related to CVD, excluding grey literature, pediatric population, and letters to the editor). Out of the 327 articles, five duplicate articles were removed, and 300 articles were filtered out after strict title screening and due to being irrelevant to the inclusion and exclusion criteria, having no full-text availability, and being irrelevant articles. So, after rigorous filtering, we are left with 22 articles, out of which four could not be retrieved, six did not pass the critical appraisal, and five did not meet the criteria for further evaluation. Thus, we were left with seven articles, all of which were large-scale randomized controlled trials and filled our criteria. These results are also demonstrated in Figure 1. The study included only adult males and females who were already predisposed to cardiovascular risk factors or had an established CVD irrespective of their diabetes status. Various SGLT2i (canagliflozin, dapagliflozin, empagliflozin, and ertugliflozin) were used in different dosages. Six out of the seven studies we chose had a positive outcome in reducing main adverse cardiac events (MACE) and cardiovascular hospitalization. The VERTIS CV trial alone showed no significant reduction in MACE or cardiovascular hospitalization. These results were obtained after a follow-up period of 2.66 years (Table 2).

**Table 2:** RCT study design RCT, randomized controlled trial; CV, cardiovascular; CVD, cardiovascular disease; MI, myocardial infarction; HFrEF, heart failure with reduced ejection fraction; LVEF, left ventricular ejection fraction; HF, heart failure; ICD, implantable cardioverter-defibrillator; MACE, main adverse cardiac event; HbA1c, hemoglobin A1c; NYHA, New York Heart Association; NT-proBNP, N-terminal pro-B-type natriuretic peptide

	Study design	Inclusion criteria	Numbers of participants	Drugs and dosage	Follow-up	Primary outcome	Conclusion
EMPA-REG (2016) [20]	Multicenter, randomized, double-blind, placebo-controlled trial	Patients with insufficient glycemic control and elevated risk of CV events	7,020	Empagliflozin 10 mg and 25 mg versus placebo	3.1 years	CV death	Lower risk of MACE in the group that took empagliflozin than in the placebo group
CANVAS (2017) [19]	Randomized, double-blind, placebo-controlled trial	Patients with type 2 diabetes mellitus with CVD	10,142	Canagliflozin 100 mg and 300 mg versus placebo	2.4 years	MACE	Patients who took canagliflozin had a decreased risk of death from CVD, MI, and stroke than the placebo group
DECLARE TIMI-58 (2018) [18]	Randomized, double-blind, placebo-controlled trial	Patients with type 2 diabetes mellitus at high risk of CV	17,160	Dapagliflozin 10 mg versus placebo	4.2 years	Heart failure hospitalization CV death	Lower rate of CV death or hospitalization for heart failure; patients with dapagliflozin showed no significant MACE rate than the placebo group
CREDENCE (2019) [17]	Randomized, double-blind, placebo-controlled trial	Patients with type 2 diabetes mellitus with HbA1c ≥ 6.5	4,401	Canagliflozin 100 mg versus placebo	2.6 years	Death from renal or CV causes and a composite of end-stage kidney disease	Patients who took canagliflozin showed a reduced risk of CV events than the placebo group
DAPA-HF (2020) [16]	Randomized, quadruple-blinded, placebo-controlled trial	Patient with symptomatic HFrEF (NYHA class II-IV) present for over two months and LVEF ≤ 40%	4,742	Dapagliflozin 5 mg and 10 mg versus placebo	1.5 years	CV death or worsening heart failure	Dapagliflozin reduced the risk of CV death irrespective of the diabetic status and decreased the risk of worsening heart failure versus the placebo group
EMPEROR-REDUCED (2020) [14]	Randomized, double-blind, placebo-controlled trial	Patients with elevated NT-proBNP, chronic HF, and patients with ICDs	3,730	Empagliflozin 10 mg versus placebo	1.3 years	Composite of heart failure hospitalization or CV death	Empagliflozin showed a lower risk of heart failure hospitalization than the placebo group, irrespective of the diabetic status
VERTIS CV (2020) [15]	Randomized, double-blind, placebo-controlled trial	Patients with type 2 diabetes mellitus atherosclerosis or peripheral vascular disease and patients on antihyperglycemic drugs	8,238	Ertugliflozin 5 mg and 15 mg versus placebo	3.5 years	MACE	Ertugliflozin showed no significant difference in MACE when compared to the placebo group

Discussion

In this systematic review, we aimed to assess the benefits of SGLT2i in patients with CVD irrespective of their diabetic status. After doing a thorough and deep online search, we selected seven studies, all of which were large-scale, randomized controlled trials with a total sample size of 55,433. We have created a table that compares the results of different trials that we have included in our study. In this review, we have described the cardiovascular outcomes of each study, the benefits of SGLT2i, and the mechanism and limitations that we faced while doing this systematic review.

Mechanism, Benefits, and Risks of SGLT2 Inhibitors

SGLT2i are a new class of drugs that have a variety of roles in the human body. They were primarily developed to help lower the blood glucose levels in patients with type 2 DM. Several types of SGLT2i have been formulated over the years. The four different types of SGLT2i that have been used in these trials mainly are empagliflozin, dapagliflozin, canagliflozin, and ertugliflozin. These drugs work primarily by preventing the glucose from being reabsorbed by the kidney’s proximal tubule, promoting natriuresis and glycosuria, and increasing the urinary excretion of glucose [21]. Recently, these drugs also started to show a cardioprotective benefit on the human body. The primary mechanism of action of how these drugs are cardioprotective is yet to be studied thoroughly to understand the mechanism.

The increased excretion of sodium by almost about 60% is thought to be a reason that decreases the afterload on the heart, thereby improving the ventricular cardiac function [22]. The DAPA-HF trial and EMPEROR-REDUCED trial have shown a reduction in the hazard ratio of 0.7 (95% confidence interval (CI): 0.58-O.85, P < 0.001) and also decreased hospital admission for heart failure [14,16].

The diuretic effect of SGLT2i is the main reason for reducing HF exacerbation and decreasing plasma volume and albuminuria (CANVAS). The benefit of the CREDENCE trial was that it had a hazard ratio of 0.61 (95% CI: 0.47-0). The 95% CI and P value, and hazard ratio for the primary outcome for each trial are shown in Table 3.

**Table 3:** RCT versus placebo RCT, randomized controlled trial; HF, heart failure; CI, confidence interval

RCT versus placebo	Hazard ratio for primary outcome	95% CI, P value
DAPA-HF (2020) [16]	0.75	0.63-0.9, 0.002
EMPEROR-REDUCED (2020) [14]	0.75	0.65-0.86, <0.001
CREDENCE (2019) [17]	0.7	0.59-0.82, 0.00001
DECLARE TIMI-58 (2018) [18]	0.83 (lower HF hospitalizations)	0.73-0.95, 0.005
VERTIS CV (2020)[15]	0.97	0.85-1.11, <0.001
EMPA-REG (2016) [20]	0.61	0.55-0.69, <0.001
CANVAS (2017) [19]	0.86	0.75-0.97, <0.001

The SGLT2i are known to express various beneficial effects on the human body beyond the glucose-lowering effects such as increasing natriuresis and diuresis, lowering blood pressure, preventing inflammation, weight loss, improving glucose control and cardiac energy, inhibiting the sympathetic nervous system, preventing adverse cardiac remodeling, preventing ischemia and reperfusion injury, increasing erythropoietin, and decreasing hyperuricemia [21]. Recent studies have also shown a possible renal protective mechanism exhibited by these drugs [23].

The DECLARE TIMI-58 trial had the largest group of people (17,000) in all the conducted trials examined and showed significantly lower hospitalizations for heart failure [18]. The CREDENCE trial was conducted on 4,401 patients, showing a decreased death from cardiovascular or renal causes. The EMPA-REG trial showed a reduction in three-point MACE (14%) and cardiovascular mortality (37%) and a reduction in hospitalization for heart failure.

Although the drugs exhibit protective mechanisms to help the body from diseases, they are also known to display many harmful risks to the human body. The major risks seen in patients who have taken SGLT2i are increased bone fractures upon taking canagliflozin, urinary tract infections, euglycemic ketoacidosis, genital mycotic infections, and hypotension, dehydration, and volume depletion. The benefits of these drugs outweigh the risks as it was shown that they exert favorable effects beyond glucose control such as reduced cardiovascular mortality and hospitalizations for heart failure [23].

SGLT2i are also known to have a minimum risk of drug-drug interactions. In these seven randomized controlled trials, these drugs were primarily used as a monotherapy drug and a placebo and showed beneficial results besides improving diabetes control by helping lower body weight, blood pressure, and uric acid levels.

Cardiovascular Outcomes

The EMPEROR-REDUCED trial had 3,730 patients; the trial included patients with diabetes, patients with a left ventricular ejection fraction of 40% or less, and class II, III, and IV heart failure. Patients were randomly assigned to receive empagliflozin or a placebo. Among the group of patients that received empagliflozin, the outcome shown was a lower risk of CVD and hospitalization due to heart failure [14].

The VERTIS CV trial was done with 8,238 patients; patients with type 2 DM and atherosclerotic CVD were randomly given 5 mg or 15 mg of ertugliflozin once a day. The outcome of ertugliflozin was not significant for MACE when compared to the placebo, indicating that the drug showed no major difference in improving the mortality and morbidity of heart failure patients [15].

The DAPA-HF trial included 4,742 patients who randomly received dapagliflozin 5 mg or 10 mg or a placebo. The trial included patients with symptomatic heart failure with reduced ejection fraction (NYHA class II-IV), patients with a left ventricular ejection fraction of <40%, and patients with type 2 DM with the addition of dapagliflozin. The patients showed a significant reduction in the risk of worsening heart failure and CVD irrespective of their diabetic status [16].

The CREDENCE trial was conducted on around 4,401 patients randomly assigned the drug canagliflozin 100 mg versus placebo. The study participants that were selected had DM with chronic kidney disease. The study results showed that kidney failure and cardiovascular events were significantly lower for patients who received canagliflozin than in the placebo group [17].

The DECLARE TIMI-58 trial included 17,160 patients with either atherosclerotic CVD with type 2 DM or atherosclerotic CVD risk factors (ASCVD). These patients randomly received dapagliflozin 10 mg or a placebo for 4.2 years. Dapagliflozin administration significantly reduced cardiovascular death and hospitalization for heart failure [18].

The CANVAS trial had a set of two trials called CANVAS and CANVAS-Renal. Both trials included 10,142 participants who had DM with established CVD. They randomly received canagliflozin 100 mg, 300 mg, or placebo, and the follow-up was for about three years. The patients treated with canagliflozin had a lower risk of cardiovascular events and a reduction of MACE [19].

The EMPA-REG trial included 7,020 patients with type 2 DM and peripheral, coronary, or cerebrovascular disease. The randomly assigned patient received empagliflozin (10 or 25 mg) or a placebo. The reported outcome of the study was that receiving empagliflozin rather than a placebo showed a significant reduction in 3P-MACE mortality.

Limitations of the study

The limitation that we encountered in the review was that we could not comment on the long-term benefits of SGLT2i, as there was not a very good follow-up in the future. In this review, we did not use articles written in any other language apart from English, and as a result, we may miss certain valuable studies that would have helped strengthen our review further. We also could not fully evaluate the adverse effects of SGLT2i. We only used studies that took place in the last five years (from 2017 to 2021). The studies we used were mainly the ones with large sample sizes; we do not have data on how much information was missed during the research time. This article did not consider other therapies or etiological factors for the treatment of heart failure apart from SGLT2i.

## Conclusions

Our systematic review was done mainly to focus on the cardiovascular benefits of SGLT2i. Six out of the seven randomized controlled trials we included in our study proved that SGLT2i affect the cardiovascular system. The studies proved that SGLT2i are well-tolerated and a safe drug in the dose range of 10, 25, and 100 mg when given to patients with CVD. These four drugs used in the trials reduced heart failure exacerbation, decreased hospitalization, and reduced MACE and cardiovascular death. Although the exact mechanism of how these drugs are beneficial is still unclear, these drugs have the potential to be considered as first-line agents for treating CVD, as they have shown favorable outcomes in trials. We still need more trials to prove the beneficial role of these drugs. We hope that long-term trials will be conducted to study the long-term benefits of SGLT2i and future research to learn about the exact mechanism of SGLT2i. We strongly believe that the drugs mentioned above have a beneficial role in reducing cardiovascular death and hospitalization by doing this review, and we strongly hope that in the future, these drugs will be used as a part of the regimen for treating CVD mainly for the benefit of the patients outweighing the risks.
